# Biting midges (Ceratopogonidae) as vectors of avian trypanosomes

**DOI:** 10.1186/s13071-017-2158-9

**Published:** 2017-05-08

**Authors:** Milena Svobodová, Olga V. Dolnik, Ivan Čepička, Jana Rádrová

**Affiliations:** 1 Department of Parasitology, Faculty of Science, Charles University, 12844 Prague 2, Czech Republic; 2 Department of Zoology, Faculty of Science, Charles University, 12844 Prague 2, Czech Republic

**Keywords:** *Culicoides nubeculosus*, *Trypanosoma bennetti*, *Trypanosoma avium*, *Trypanosoma culicavium*, Life-cycle, Transmission, Host specificity, Avian parasite

## Abstract

**Background:**

Although avian trypanosomes are widespread parasites, the knowledge of their vectors is still incomplete. Despite biting midges (Diptera: Ceratopogonidae) are considered as potential vectors of avian trypanosomes, their role in transmission has not been satisfactorily elucidated. Our aim was to clarify the potential of biting midges to sustain the development of avian trypanosomes by testing their susceptibility to different strains of avian trypanosomes experimentally. Moreover, we screened biting midges for natural infections in the wild.

**Results:**

Laboratory-bred biting midges *Culicoides nubeculosus* were highly susceptible to trypanosomes from the *Trypanosoma bennetti* and *T. avium* clades. Infection rates reached 100%, heavy infections developed in 55–87% of blood-fed females. Parasite stages from the insect gut were infective for birds. Moreover, midges could be infected after feeding on a trypanosome-positive bird. Avian trypanosomes can thus complete their cycle in birds and biting midges. Furthermore, we succeeded to find infected blood meal-free biting midges in the wild.

**Conclusions:**

Biting midges are probable vectors of avian trypanosomes belonging to *T. bennetti* group. Midges are highly susceptible to artificial infections, can be infected after feeding on birds, and *T. bennetti*-infected biting midges (*Culicoides* spp.) have been found in nature. Moreover, midges can be used as model hosts producing metacyclic avian trypanosome stages infective for avian hosts.

## Background

Trypanosomes (Kinetoplastea: *Trypanosoma*) are dixenous protozoan parasites that occur in the blood of vertebrates and are transmitted by blood-sucking invertebrates. Avian trypanosomes, although being widespread, are neglected due to their low economic importance, yet they are used as model parasites in the research of host-parasite interactions. Based on molecular studies, avian trypanosomes have been shown to form three distinct evolutionary groups, each consisting of several lineages [1–3]. Although the life-cycles of several lineages of avian trypanosomes belonging to groups B and C have been clarified (see below), many of them remain unknown, including the entire *T. bennetti* (*s.l*.) group A.

Previously, we have described or confirmed life-cycles of several avian trypanosome species which differ in vectors and localisation in the gut. Two of them, *Trypanosoma* cf. *avium* and *T. corvi*, have mature infections localised in the hindgut of the insect [4]. While *Trypanosoma* cf. *avium* is transmitted by blackflies (*Simulium* spp.) by ingestion or via conjunctiva [5, 6], *T. corvi* is transmitted by ingestion of hippoboscid flies (*Ornithomyia avicularia*) [7, 8]. Mature stages of *T. culicavium* are localised on the stomodeal valve of *Culex* mosquitoes, suggesting inoculative transmission; however, birds get infected by ingestion of the vector as well [2].

The vector of *Trypanosoma bennetti* (and related lineages of avian trypanosomes) remains unknown. *T. bennetti* was originally isolated from American Kestrel (*Falco sparverius*), and described on the basis of the morphology of culture forms, lectin binding patterns, and isoenzyme analysis; no bloodstream trypomastigote has been found, and the parasites did not establish infection in *Aedes aegypti* that fed on infected birds [9]. Later, strains belonging to *T. bennetti* group were found in European passerine birds as well as in raptors, and isolation from nestlings and yearlings confirmed local transmission [1]. The *T. bennetti* parasites were never found in blood-sucking Diptera, including numerous biting midges trapped while attacking raptor nestlings [4, 10].

Bennett [5] was the first to study the role of ceratopogonids as vectors of avian trypanosomes. Trypanosomes multiplied in the gut of *Culicoides sphagnumensis, C. stilobezzioides*, and *C. crepuscularis* that had fed on infected birds but were lost at defecation. However, parasites taken from these midges guts were infective after injection to a Java Sparrow (*Lonchura oryzivora*); therefore, the author concluded that midges are occasional but poor vectors. On the other hand, an unspecified number of laboratory-bred *C. nubeculosus* was infected by feeding on Blossom-headed Parakeet (*Psittacula roseata*), and trypanosomes were transmissible to a parakeet by intraperitoneal injection of crushed infected midges [11]. The donor bird was not infective for *Aedes aegypti* and *Anopheles stephensi*. Unfortunately, in these early studies trypanosomes were not molecularly characterised; therefore, their phylogenetic position and species identity remain unclear. In this study, we focus on the potential of biting midges to transmit avian trypanosomes from distinct lineages, with emphasis to *T. bennetti* clade which has no known vector.

## Methods

### Parasite strains and culture

The trypanosome strains used in this study were our isolates: APO7 was isolated from a Lesser Spotted Eagle (*Aquila pomarina*) nestling in Eastern Slovakia (AAQU/SK/2000/APO7); PAS23 from an adult Yellowhammer (*Emberiza citrinella*) (AEMB/CZ/2002/PAS23); CUL1 and CUL6 from a female of *Culex pipiens* (ICUL/CZ/1998/CUL1, ICUL/CZ/2000/CUL6); BUT15 from Common Buzzard (*Buteo buteo*) nestling (ABUT/CZ/1999/BUT15); all from Milovice forest, South Moravia, Czechia. These isolates represented three major avian trypanosome clades; APO7 belongs to *T. bennetti* (*s.s*.), PAS23 is a sister clade to *T. bennetti*, CUL1 and CUL6 are *T. culicavium* (*s.s*.), and BUT15 is *T. avium* (*s.s*.) (for a detailed phylogenetic study see [1]).

Trypanosomes were cultivated on rabbit blood agar (SNB-9) in flat tubes, with the original overlay (*T. avium*) or RPMI 1640/Schneider Drosophila Medium 1:1, supplemented with 10% FCS/2% sterile human urine/50 μg/ml amikacin (R + S).

### Insect experimental infections

Experimental *Culicoides nubeculosus* biting midges given from CIRAD, Montpellier, France, originated from the Pirbright Institute (Pirbright, UK); mosquitoes *(Culex quinquefasciatus*) from our laboratory colony; *Aedes aegypti* from a colony kept in Institute of Parasitology, Biology Center CAS, in České Budějovice. Females were exposed to parasites by feeding through a chick skin membrane on heat-inactivated rabbit blood (Bioveta) containing 7–14-day-old culture of 5 × 10^6^–2 × 10^7^ parasite cells/ml. Three independent feeding experiments were performed with *C. nubeculosus* and APO7, two with both CUL 6 and BUT15. Fed females were separated immediately after blood-feeding, kept at 20 °C, ambient humidity, and supplemented with 50% sucrose solution on a cotton pad. Dissected guts were examined under a light microscope for infection status, intensity of infection, and parasite localization.

### Experimental birds

Canaries (*Serinus canaria*) were bought in a pet shop. Before inoculation, they have been screened for trypanosome infection by blood culture; all of them were negative. The Canaries were inoculated with 7–20 positive guts (whole insects in one case) using different modes (see Table 1) and checked at 7 or 14-day intervals for infection status. In positive cases, parasite identity was confirmed by sequencing of the SSU rRNA gene.

**Table 1:** Morphometry of putative metacyclic trypomastigotes in *Culicoides nubeculosus* biting midges gut

Trait^a^	*T. bennetti* (*s.s*.) APO7 (*n* = 50)	*T. bennetti* (*s.l*.) PAS23 (*n* = 55)	*T*. cf. *avium* BUT15 (*n* = 33)
Length	19.8 ± 0.4 (14.6–24.7)	16.7 ± 0.5 (11.5–21.8)	114.0 ± 0.3 (12.0–17.8)
Width	0.9 ± 0.1 (0.6–1.5)	1.1 ± 0.1 (0.5–1.7)	1.4 ± 0.1 (1.0–2.3)
Flagellum	6.9 ± 0.2 (4.5–11.2)	6.5 ± 0.3 (3.1–9.1)	5.7 ± 0.2 (3.9–8.9)
KN	3.3 ± 0.1 (1.8–4.7)	2.0 ± 0.1 (1.1–5.1)	0.9 ± 0.1 (0.6–1.4)
PK	3.4± 0.1 (1.7–5.3)	2.7 ± 0.1 (1.2–3.8)	2.4 ± 0.1 (1.2–4.2)
PN	6.6 ± 0.2 (4.4–8.7)	4.5 ± 0.2 (2.4–6.8)	2.5 ± 0.1 (1.5–4.7)

*Length* total length of the cell without free flagellum, *Width* width of body through centre of nucleus, *Flagellum* free flagellum, *KN* kinetoplast to centre of nucleus, *PK* posterior end to kinetoplast, *PN* posterior end to centre of nucleus

^a^Values in μm, average ± SE, range is given in parentheses

### Trypanosome diagnostic sampling

Diagnostic cultivation of trypanosomes was done in glass vials as described previously [4] Briefly, 10–20 μl of blood from the metatarsus vein articulation (*vena metatarsalis plantaris superficialis media*) was diluted in a tuberculine syringe with culture medium R + S (250 μl) and seeded on blood agar.

### Transmission of trypanosomes from canaries to midges

To test if midges can be infected by feeding on the trypanosome-positive bird, they were allowed to feed on canaries experimentally infected with *T. avium* strain BUT15. Since the midges did not feed in darkness, canaries had to be held by hand in a net with midges to prevent aversive behaviour of the birds. They were exposed for 15 min; the interval between repeated exposures was at least four days. Fed midge females were separated immediately after the experiment, and their survival was checked daily. Dead females were analysed by PCR (see below), survivors were dissected seven days after feeding.

### Diagnostic PCR in midges and sequencing of the positive cultures

DNA from individual biting midges or cultures was extracted using the High Pure PCR Template Preparation Kit (Roche Diagnostics, Mannheim, Germany), according to the manufacturer’s instructions. For parasite identification, trypanosome SSU rRNA gene was amplified using a specific nested PCR. The first amplification round consisting of 35 cycles was performed in a final volume of 20 μl of PCR mix (TopBio, Prague, Czech Republic) with S762 and S763 primers [12]. For the next step consisting of 35 amplification cycles, 1 μl of amplified product was used as a template with nested primers TR-F2 and TR-R2 [13]. Electrophoresis-positive bands were cut from the gel, purified using a High Pure PCR Product Purification Kit (Roche Diagnostics) according to the manufacturer’s instructions, and sequenced in the Core Facility of Faculty of Science. The newly determined sequences were 469–779 bp long.

BLAST indicated that all four newly determined sequences belonged to the avian trypanosome group A as defined in [1] (i.e. they appeared closely related to *T. bennetti*). To examine their phylogenetic position more thoroughly, we carried out a phylogenetic analysis. A data set containing SSU rRNA gene sequences was created, consisting of four newly determined sequences and 27 sequences of trypanosomes belonging to the group A, retrieved from GenBank. The sequence of *T. irwini* was used as an outgroup. The sequences were aligned using the MAFFT method [14] using the MAFFT 7 server (http://mafft.cbrc.jp/alignment/server/) with the G-INS-i algorithm at default settings. The alignment was manually edited using BioEdit 7.0.9.0 [15]. The final data set of unambiguously aligned characters consisted of 2163 positions and is available upon request from the corresponding author. The phylogenetic tree was constructed by the maximum likelihood method in RAxML 8.0.0 [16] under the GTRGAMMAI model. Node support was assessed by an analysis of 1000 bootstrap data sets.

### Light microscopy

Blood smears and parasites from dissected midge guts were fixed on slides with methanol and stained with Giemsa stain, Fluka. Cell morphotypes from the guts were photographed at 1000× magnification with CCD camera DP70 using Olympus BX51 microscope and bright-field optics and measured using the Image-J software. The morphometric features studied included (i) total length of the cell without free flagellum; (ii) width of body through centre of nucleus; (iii) free flagellum length; (iv) distance between kinetoplast to centre of nucleus; (v) distance from posterior end to kinetoplast; (vi) distance from posterior end to centre of nucleus.

### Scanning electron microscopy

Dissected guts positive for parasites were fixed in 2.5% glutaraldehyde in 5 mM HCl, 0.1 M cacodylate buffer for 24 h at 4 °C. Samples were post-fixed in 2% osmium tetroxide in the same buffer for 2 h at room temperature. After dehydration in a graded ethanol series, the guts were critical-point air-dried, sputter coated with gold in a Polaron coater and examined using a JEOL 6380 LV scanning electron microscope.

### Insect collection and identification

Biting midges were trapped using CDC light traps (JW Hock company, Gainesville, Florida) baited with dry ice in 2015–2016 in Milovice game preserve. They were sorted according to species [17, 18], and screened by nested PCR (see above) for the presence of kinetoplastids in groups of ten or less specimens.

## Results

### Experimental infection of *C. nubeculosus* biting midges

**Fig. 1:**
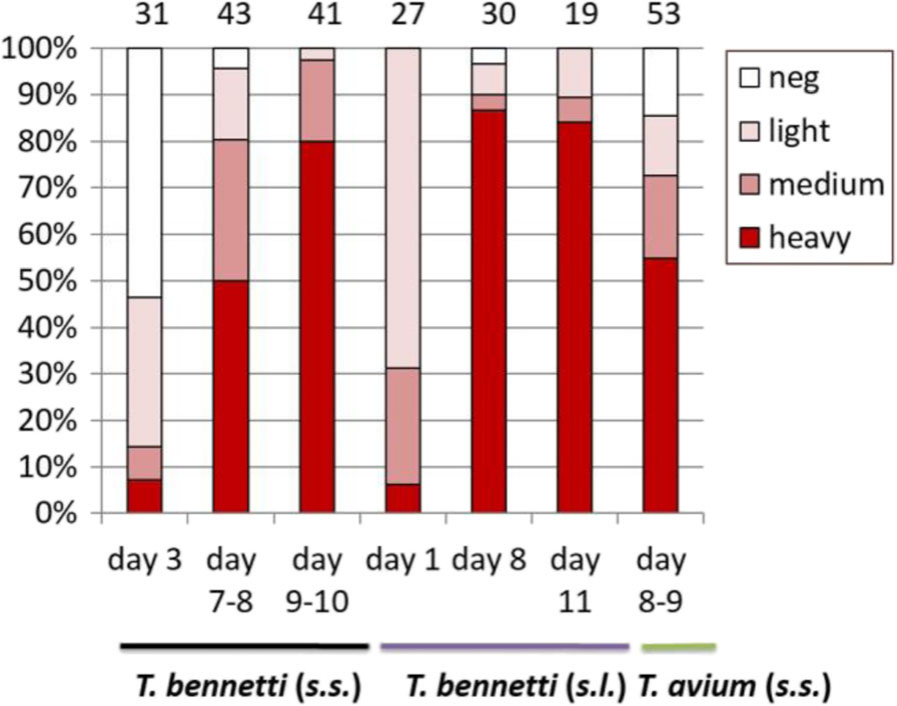
Infection rates and intensities in biting midges *Culicoides nubeculosus* membrane-fed on *Trypanosoma bennetti* and *T. avium.* Infection intensities: low - 1–100 parasites; medium - 100–1000 parasites; heavy- > 1000 parasites per gut. Numbers of dissected females shown above the columns

*C. nubeculosus* was highly susceptible to several strains of avian trypanosomes. After defecation, over 90% of individuals were infected with *T. bennetti* strain APO7; heavy infections (more than 1000 parasites per gut) were found in 50% of individuals on day 8–9, and in 80% on day 9–10 (Fig. 1). High susceptibility has been found for PAS23 strain as well, which is closely related to *T. bennetti* (*s.s*.). Nearly 100% of individuals were infected, and heavy infections occurred in 87% of individuals after defecation (day 7–8; Fig. 1).

The permissiveness of *C. nubeculosus* was not restricted to *T. bennetti* clade but included *T.* cf *avium* as well. After feeding on strain BUT15, 85% of midges were infected, and heavy infections were found in 55% on day 7–10 (Fig. 1).

On the other hand, after feeding on *T. culicavium* CUL1, none of 34 dissected *C. nubeculosus* was infected (day 7–11). Control *Cx. quinquefasciatus* were infected in 27% (*n* = 26). Feeding on *T. culicavium* CUL6 did not result in infection either (day 8–9, *n* = 11) while controlling *Cx. quinquefasciatus* mosquitoes were infected in 61% (*n* = 28).

*Aedes aegypti* was tested as a potential vector of *T. bennetti* as well. None of the mosquitoes that fed on blood with *T. bennetti* APO 7 and were dissected on days 15–28 was found infected (*n* = 90).

*Culex quinquefasciatus* did not support the infection with APO7 either; out of 67 individuals dissected on days 17–22, none became infected.

### Localisation and morphology of parasites in midges

**Fig. 2:**
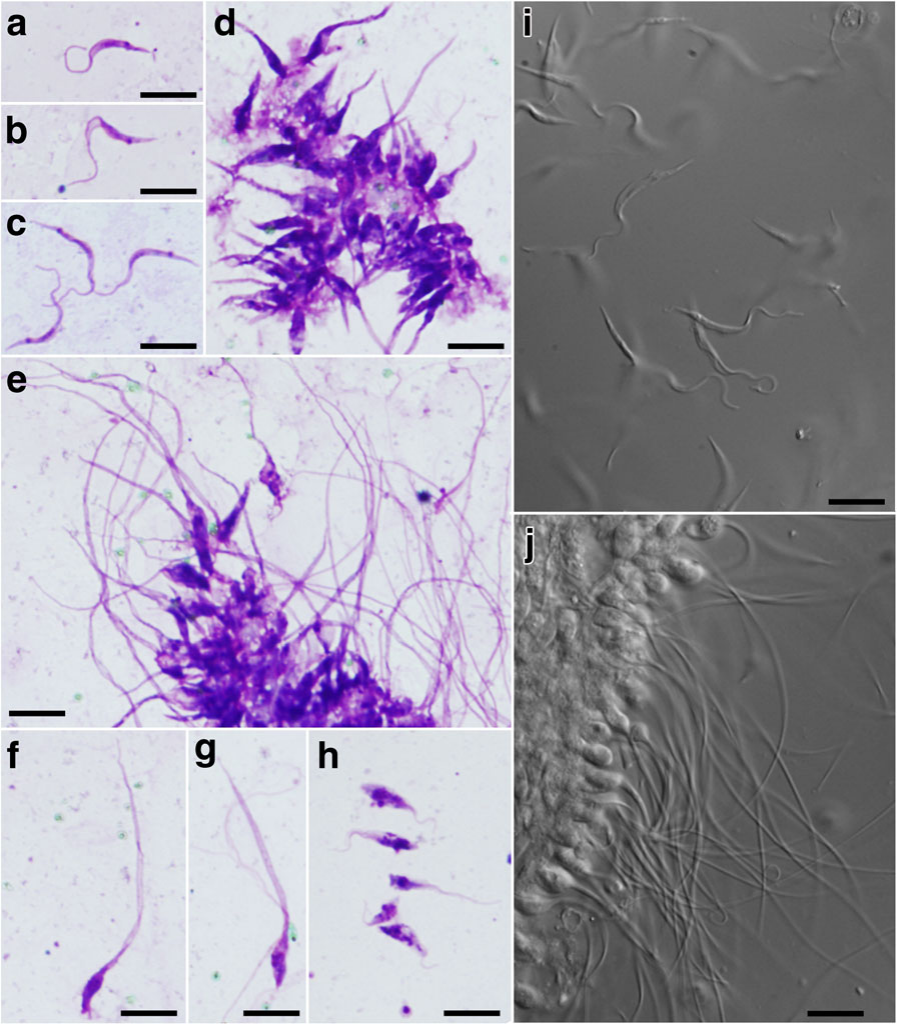
Light microscopy of trypanosome morphotypes in biting midges gut, Giemsa stained (**a**-**h**) and live (**i**, **j**). Trypomastigotes of *T. bennetti* (*s.l*.) strain PAS 23 (**a**, **b**), and *T. bennetti* (*s.s*.) strain APO7 (**c**, **i**); rosettes of haptomonads (**d**) and haptomonads with long cytoplasmic protrusions (**e**, **j**), individual haptomonads (**f**, **g**), all APO7; metacyclic trypomastigotes of *T. avium* BUT15 (**h**). Bright-field (**a**-**h**), differential interference contrast (**i**, **j**). *Scale-bars*: 10 μm

**Fig. 3:**
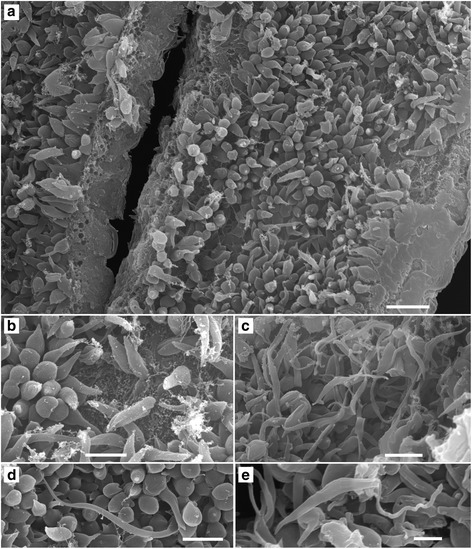
Scanning electron microscopy of *T. bennetti* (*s.s*.) APO7 in the gut of biting midge *C. nubeculosus*. Note the massive infection of haptomonads covering the gut (**a**); a detailed view of haptomonads attached to the epithelium (**b**); long cytoplasmic protrusion of some haptomonads (**d**); free-swimming epimastigotes (**c**, **e**). *Scale-bars*: **a**, 10 μm; **b**-**d**, 5 μm; **e**, 2 μm

The primary localisation of strains belonging to *T. bennetti* clade is the abdominal midgut. Nearly 100% of infected midges harboured parasites there; about half of them had parasites in the thoracic midgut as well. No parasites were found on the stomodeal valve, and rarely they were seen in the hindgut. On the contrary, infections of *T. avium* BUT15 were localised in the hindgut and rectal ampulla. Free-swimming forms were trypomastigotes, presumably metacyclic infective for the avian hosts (Table 2). Parasites from the *T. bennetti* clade were polymorphic; besides free parasites, the gut epithelium was densely covered by attached forms (haptomonads) with the tapered posterior part, sometimes reaching 80 μm in length (Figs. 2, 3).

**Table 2:** Results of trypanosome cultivation from experimental canaries inoculated perorally (po), transconjuctivally (tc) or subcutaneously (sc)

Bird	Strain	Dose	Infection route	Result	Day first positive	Day last positive	Day last checked
1	APO7	20 guts	po	neg			74
2	APO7	20 females	po	neg			166
3	PAS23	7 guts	tc	neg			117
4	PAS23	9 guts	sc	pos	6	27	209
5	APO7	11 guts	sc	pos	2	102	162
6	APO7	12 guts	sc	“relaps”	168	168	223
7	APO7	12 guts	po	neg			222
8	BUT15	10 guts	po	pos	19	159	247

### Experimental infections of canaries

All three strains of avian trypanosomes used in our experiments were infective for canaries. Parasites were detectable in blood as early as two days after inoculation. *T. bennetti* (*s.l*.) (strains APO7 and PAS23) were able to infect canaries only after subcutaneous inoculation of midge guts harbouring trypanosomes. Birds inoculated perorally, or transconjunctivally remained negative. *T. avium*, on the other hand, was infective after peroral inoculation (Table 1).

One of the birds inoculated with *T. bennetti* APO7 was positive between days 6 and 27, then remained negative until the end of sampling. Another one, however, was positive on day 2, then on day 49, and again on day 82, while sampling in between remained negative. Another individual was positive only on a single occasion on day 168 after inoculation. A bird inoculated with *T. avium* BUT15 developed detectable parasitaemia after peroral inoculation that lasted 140 days (Table 1).

### Infection of *C. nubeculosus* by feeding on canaries (xenodiagnosis)

Out of 20 females that had fed on canaries positive for *T. avium* BUT15, 25% were positive. Most of the females did not survive until day 7, and these were tested by PCR (days 3–6 after feeding). One out of four females dissected on day seven harboured mature infection with parasites localised in the hindgut. The identity of the parasites was confirmed by sequencing of the SSU rRNA gene; the obtained sequences were identical to the sequences of the strains used to infect the host bird.

### *Culicoides* spp. naturally infected with *T. bennetti* (*s. l*.)

**Fig. 4:**
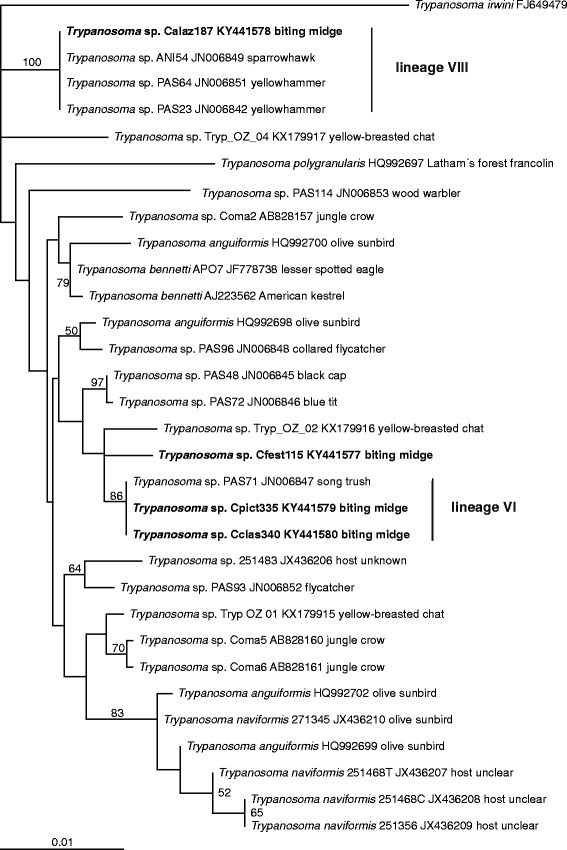
Phylogenetic tree of the group A of avian trypanosomes (*T. bennetti* clade, see [1]) based on the SSUrRNA gene sequences. The tree was constructed by the maximum likelihood method in RAxML (GTRGAMMAI model). The values at branches represent statistical support in bootstrap values (RAxML). Support values below 50 are not shown. New sequences in bold. The scale-bar represents 10 changes per 100 positions

Among 1184 trapped midges, we found four individuals naturally infected by kinetoplastids. BLAST search the SSU rRNA gene demonstrated that all four specimens were infected by avian trypanosomes from the *T. bennetti* (*s.l*.) group [1]. This was confirmed by a phylogenetic analysis of the group A, which also showed that the sequences branch at three different positions (Fig. 4): (i) the sequence of *Trypanosoma* sp. Calaz187 (from *C. alazanicus*, GenBank KY441578) was identical with sequences of strains ANI54, PAS64, and PAS23; (ii) sequences of *Trypanosoma* sp. Cpict335 and *Trypanosoma* sp. Cclas340 (from *C. pictipennis* and *C. clastrieri*, resp., GenBank KY441579, KY441580) were identical with each other and were closely related to the sequence of strain PAS71, differing from it by a single nucleotide; and (iii) *Trypanosoma* sp. Cfest115 (from *C. festivipennis*, GenBank KY441577) formed a branch closely related to a clade formed by PAS71, Cpict335, and Cclas340. According to BLAST, it was most similar to the sequence of strain PAS71 (99% identity).

## Discussion

Vectors of avian trypanosomes and their transmission ways are still insufficiently known. In our previous studies of ornithophilic biting midges, we have not detected any avian trypanosomes [10], despite the fact that midges appear notoriously on the list of avian trypanosome vectors. *T. bennetti* was described in 1986 [9], yet, its vector remained unknown. During an extensive study of ornithophilic blood-sucking Diptera as avian trypanosome vectors, no parasite belonging to the *T. bennetti* group has ever been found in the insects examined by microscopy and culture; namely in mosquitoes, black flies, and hippoboscids [4]. Therefore, we decided to test the interaction between these “orphans”. First, midges have been suggested as trypanosome vectors in earlier studies [5, 11]; moreover, biting midges of genus *Forcipomyia* have been recently identified as probable vectors of *Leishmania enriettii* (*s.l*.) in Australia [19], proving their potential as vectors of dixenous kinetoplastids. Australian field findings were supported by recent experiments testing the vectorial capacity of biting midges *C. nubeculosus* and *C. sonorensis* for *L. enriettii* (*s.l*.). Only *C. sonorensis* became infected, with maximum infection rates of 38%, and heavy infections in approximately 3% of fed females [20]. With a similar infectious dose of *T. bennetti* (*s.l*.), we demonstrate an infection rate of 100% in late infections, with heavy infections in 80% of *C. nubeculosus*; moreover, *C. sonorensis* developed 87% of heavy late stage infections with *T. bennetti* as well (data not shown). Biting midges thus have a high capacity to sustain the development of *T. bennetti* group.

The diagnostic method can influence the outcome of parasite screening. In our previous study on ornithophilic biting midges, dissections of 2493 guts of midges belonging to seven *Culicoides* species revealed 1.4% prevalence of kinetoplastids. From 36 positive guts, eight strains have been established that belonged to monoxenous trypanosomatids [10, 21, 22]. Potentially, some of the unsuccessful cultures might belong to *T. bennetti* clade. However, their insect stages morphotypes were much smaller (cell length ~ 10 μm; Svobodová, unpublished) than *T. bennetti* in *C. nubeculosus* (~20 μm), and resembled the monoxenous parasites that have been established in cultures and characterized molecularly, with only a single exception (cell length ~17 μm). This suggests that the parasites in our earlier studies indeed were not trypanosomes.

In the field part of our research, we have found biting midges naturally infected by trypanosomes belonging to the *T. bennetti* group, namely, *T. bennetti* (*s.l*.) lineage VIII in *C. alazanicus*, Lineage VI in *C. pictipennis* and *C. clastrieri,* and a new lineage related to lineage VI in *C. festivipennis*. The most closely related sequences, ANI54, PAS64, PAS23, and PAS71, were obtained from naturally infected avian hosts, yellowhammers (*Emberiza citrinella*), a song thrush (*Turdus philomelos*), and a sparrowhawk (*Accipiter nisus*); passerines were trapped at the same locality as biting midges confirming local transmission since yellowhammers are residents. These findings are in agreement with the former finding of a single specimen of *C. pictipennis* positive for *T.* cf. *bennetti* [23]. PCR positive specimens did not contain visible blood remnants, confirming that biting midges might serve as natural vectors of *T. bennetti* group in central Europe. However, without experimental confirmation of vectorial capacity, PCR positivity has to be taken with caution since *C. nubeculosus* was shown to remain PCR positive for *Leishmania* parasites (that are not transmitted by midges) even after the blood meal has been digested and defecated [24]. Prevalences of avian trypanosomes in other dipteran genera of avian trypanosome vectors usually ranged between 4 and 8% [4], while in our recent sample of wild-caught midges it was lower (0.3%); however, in other dipteran genera tested simultaneously, not a single infection with *T. bennetti* has been found (Rádrová et al., unpublished), which supports the specific role of biting midges as its vectors.

In our study, the infectivity of *T. bennetti* cells at the late stage of vector infection has been proven on experimental canaries, although only after subcutaneous inoculation. Since the infections were localised in the abdominal midgut of the insects, the inoculative transmission seems improbable in nature. In earlier studies that used unspecified avian trypanosomes, only one-third of inoculated canaries turned positive, and no transmission occurred transconjunctivally [25]. Failure of other inoculation ways might be explained by some innate resistance of canaries to natural modes of transmission.

A high susceptibility of *C. nubeculosus* to *T. avium* (*s.s*.), which is naturally transmitted by blackflies, and the ability of parasites from midges to infect birds, reveals that trypanosomes do not have a strict vectorial specificity. Indeed, trypanosome lineages with known vector-parasite associations were isolated from hippoboscid flies besides their usual vectors [1]. Hippoboscids are permanent parasites that feed daily, creating a nutritious culture medium in their gut; therefore, trypanosome presence in hippoboscids has to be interpreted with caution. However, *T. avium* mature infections have been described in naturally infected *Lutzomyia caballeroi* as well [26]. Avian trypanosomes, therefore, seem to be not strictly vector-specific; nevertheless, some degree of specificity still exists since in our experiments, *T. culicavium* was not infectious to biting midges; moreover, *T. bennetti* infected neither *Cx. pipiens* nor *Ae. aegypti*. The latter has been found to support the development of some avian trypanosomes, but localisation of the infectious stages in the hindgut suggests that these belonged to *T. avium* (*s.l*.) [1, 5].

Experimental inoculations of canaries revealed mixed results. Interestingly, in one of the canaries inoculated with *T. bennetti* (*s.s*.) trypanosomes have been detected only after 168 days, possibly due to stress induced by breeding, moving to a new site, naturally prolonged light period, or all in concordance. The appearance of avian trypanosomes in the blood due to stress has been shown using naturally infected blackcaps (*Sylvia atricapilla*) [27]. Trypanosomes thus may remain undetected for a long period, but nevertheless, the bird potentially develops detectable parasitaemia. In the other individuals, the duration of detectable parasitaemia was also rather unpredictable, lasting between 21 and 100 (140 for *T. avium*) days. An unpredictable pattern of detectable parasitaemia has been found for *Trypanosoma* spp. in canaries in earlier studies as well [25].

We demonstrated the competence of biting midges to acquire trypanosome infection in a natural way by feeding midges on infected canaries; 25% of those became infected. Our experimental species *C. nubeculosus* is considered to be mammalophilic, feeding on a large spectrum of mammals [28]; however, the species that we have found naturally infected with avian trypanosomes have repeatedly been found to feed on birds, and are considered ornithophilic or at least opportunistic [29–31]. The findings of midges infected with avian trypanosomes further confirm not only their association with avian hosts but also their potential to function in the transmission cycle.

## Conclusions

Biting midges are probable vectors of avian trypanosomes belonging to *T. bennetti* group, based both on their high susceptibility to artificial infections, and on findings of *T. bennetti*-infected biting midges (*Culicoides* spp.) in nature. The susceptibility of midges to *T. avium* but not *T. culicavium* group suggests that distinct avian trypanosome lineages differ in their ability to develop in alternative vectors. Midges can also be used as model hosts producing metacyclic avian trypanosome stages infective for avian hosts.
